# Voice break in boys—temporal relations with other pubertal milestones and likely causal effects of BMI

**DOI:** 10.1093/humrep/dez118

**Published:** 2019-07-26

**Authors:** A S Busch, B Hollis, F R Day, K Sørensen, L Aksglaede, J R B Perry, K K Ong, A Juul, C P Hagen

**Affiliations:** 1 Department of Growth and Reproduction, Rigshospitalet, University of Copenhagen, Copenhagen O, Denmark; 2 International Center for Research and Research Training in Endocrine Disruption of Male Reproduction and Child Health, Rigshospitalet, University of Copenhagen, Copenhagen O, Denmark; 3 MRC Epidemiology Unit, Institute of Metabolic Science, University of Cambridge School of Clinical Medicine, Cambridge Biomedical Campus Box, Cambridge, UK; 4 Department of Paediatrics, University of Cambridge School of Clinical Medicine, Cambridge Biomedical Campus, Cambridge, UK

**Keywords:** voice break, puberty, male, BMI, causal, Mendelian randomization

## Abstract

**STUDY QUESTION:**

How is timing of voice break related to other male pubertal milestones as well as to BMI?

**SUMMARY ANSWER:**

We provide a comprehensive temporal analysis of male pubertal milestones, including reproductive hormone dynamics, confirm voice break as a late milestone of male puberty and report a likely causal relationship between higher BMI and earlier age at voice break in men.

**WHAT IS KNOWN ALREADY:**

Voice break represents a late pubertal milestone and recalled age at voice break is frequently used in epidemiological studies as a measure of puberty. In contrast, clinical studies use mainly testicular enlargement and/or genital tanner stage as the marker of pubertal onset. However, neither correlation of pubertal milestones nor reproductive hormone dynamics have been assessed in detail previously. Further, although BMI and puberty timing are known to be closely linked, cause and effect between these traits are not known.

**STUDY DESIGN, SIZE, DURATION:**

The study included a population-based mixed cross-sectional and longitudinal cohort (2006–2014, COPENHAGEN Puberty Study) of 730 healthy Danish boys. Data for 55 871 male research participants from the 23andMe study were obtained, including genome-wide single nucleotide polymorphism data and age at voice break.

**PARTICIPANTS/MATERIALS, SETTING, METHODS:**

We performed a detailed evaluation of pubertal milestones and reproductive hormone levels (study population 1). A Mendelian randomization (MR) approach was used to determine the likely causal link between BMI and timing of voice break (study population 2).

**MAIN RESULTS AND THE ROLE OF CHANCE:**

Voice break occurred at mean age 13.6 (95% CI: 13.5–13.8) years. At voice break, mean (95% CI) testosterone levels, LH levels and bi-testicular volume were 10.9 (10.0–11.7) nmol/L, 2.4 (2.2–2.5) IU/L and 24 (23–25) mL, respectively. Voice break correlated moderately strongly with timing of male pubertal milestones, including testicular enlargement, gonadarche, pubarche, sweat odor, axillary hair growth and testosterone above limit of detection (*r^2^* range: 0.43–0.61). Timing of all milestones was negatively associated with age-specific BMI (all *P* ≤ 0.001). MR analyses inferred likely causal effects of higher BMI on earlier voice break in males (−0.35 years/approximate SD, *P* < 0.001).

**LIMITATIONS, REASONS FOR CAUTION:**

Participation rate of the population-based cohort was 25%. Further, boys that were followed longitudinally were examined approximately every 6 months limiting the time resolution of pubertal milestones. Using adult BMI as exposure instead of prepubertal BMI in the MR analysis and the known inaccuracies of the testosterone immunoassay at low testosterone levels may be further limitations.

**WIDER IMPLICATIONS OF THE FINDINGS:**

We provide valuable normative data on the temporal relation of male pubertal milestones. Further, the likely causal relationship between BMI and puberty timing highlights the importance of preventing obesity in childhood.

**STUDY FUNDING/COMPETING INTEREST(S):**

This work was supported by Danish Agency for Science, Technology and Innovation (09-067 180); Danish Ministry of the Environment, CeHoS (MST-621-00 065); Capital Region of Denmark (R129-A3966); Ministry of Higher Education and Science (DFF-1331-00 113); Innovation Fund Denmark (InnovationsFonden, 14-2013-4); The International Center for Research and Research Training in Endocrine Disrupting Effects of Male Reproduction and Child Health. B.H., F.R.D., J.R.B.P. and K.K.O. are supported by the Medical Research Council (MC_UU_12015/2). The 23andMe study is supported by the National Human Genome Research Institute of the National Institutes of Health (R44HG006981). Members of the 23andMe Research Team are employees of 23andMe, Inc. and hold stock or stock options in 23andMe.

**TRIAL REGISTRATION NUMBER:**

NCT01411527

## Introduction

Puberty is characterized by the reactivation of the hypothalamic–pituitary–gonadal (HPG) axis after childhood leading to physical changes and sexual maturation. The timing of puberty is linked to a number of later health outcomes, such as lifetime incidence of obesity, type 2 diabetes, hypertension and cancer (Day *et al*., 2015b; Hollis *et al*., 2018).

Clinical evaluation of genital development (gonadarche) or, as a more objective and quantifiable measure, testicular enlargement equal to or above 4 mL, represents the gold standard in the clinical evaluation of male pubertal progression. However, timing of other pubertal milestones, for example voice break, is frequently used as a measure for male puberty (Andersen, 1968; Harries *et al*., 1997; Ong *et al*., 2012; Yousefi *et al*., 2013). This counts particularly for epidemiological studies including studies on secular trends of pubertal timing (Juul *et al*., 2007; Brix *et al*., 2019) and large-scale genome-wide association studies (GWAS; Day *et al*., 2015a; Hollis *et al*., 2018) where clinical data on genital development are usually not available. Following laryngeal growth, voice break, i.e. the abrupt decrease in the fundamental voice frequency, occurs when the vocal cords lengthen (Kahane, 1982). In boys, additional clinical signs of ongoing sexual maturation include pubic and axillary hair development, growth spurt and sweat odor. Despite this frequent use of secondary pubertal milestones as a measure for male puberty, the temporal relation between timing of male pubertal milestones, particularly timing of voice break, is not fully elucidated. Further, while the association between BMI and timing of puberty in girls appears to be consistent across studies (Ahmed *et al*., 2009), the association between BMI and timing of puberty in boys remains debated: while some studies find an inverse correlation of BMI and male puberty timing (Sandhu *et al*., 2006), other studies report delayed puberty in obese boys (Lee *et al*., 2016). In addition, although BMI and puberty timing are known to be closely linked, cause and effect between these traits are still debated (Reinehr and Roth, 2018). A recently developed genetic tool, i.e. two-sample Mendelian randomization (MR), using genetic variants as an instrumental variable enables to estimate a causal link between exposure and outcome under some causal assumptions (Burgess *et al*., 2013).

In the present study, we evaluate timing as well as temporal relation of different male pubertal milestones, including reproductive hormones, in healthy boys. Further, we study the association and likely causal relation between BMI and pubertal timing in boys using both observational and large-scale genetic data.

## Materials and Methods

### Study populations

Participants were recruited as part of the COPENHAGEN Puberty Study (Aksglaede *et al*., 2009b; Sørensen *et al*., 2010; ClinicalTrials.gov ID: NCT01411527), a population-based cohort study of healthy Danish children and adolescents. Detailed information about the study has been given previously (Sørensen *et al*., 2010). In brief, the study is a mixed cross-sectional (Cohort A; aged 6.1–21.9 years) and longitudinal (Cohort B; age at baseline: 5.8–15.6 years) study conducted at 10 schools in the Copenhagen area from 2006 to 2014. In total, 3101 boys were invited to participate in the study with an overall participation rate of ~25%. Of 799 healthy boys with available clinical pubertal staging, we excluded 69 boys due to non-Danish origin (*n* = 67) or history of severe chronic illness (*n* = 2). After exclusion, data from 730 boys (cross-sectional study *n* = 637; longitudinal study *n* = 93) comprising 1509 visits were analysed.

### Clinical examination

Pubertal examinations were performed by one of three male doctors who were trained by an experienced pediatric endocrinologist (A.J.) to evaluate pubertal development. Clinical examinations included pubertal staging of genital development according to Tanner’s classification (genital and pubic hair development by visual assessment) and testicular volume (Marshall and Tanner, 1970). Testicular volume to the nearest millilitre was assessed by palpation using a Prader orchidometer. Testicular volume of 4 mL and above (at least one testis) was considered a marker of testicular enlargement, ≥G2 (scrotal enlargement and change in texture of the scrotal skin) defined gonadarche and ≥PH2 (sparse development of pigmented hair at the base of penis) defined pubarche. Evaluation of axillary hair growth was performed by inspection. Sweat odor was recorded as a binary variable (yes/no) by self-reported characteristic change in body odor and by assessment during examination: in case of incongruence, the latter was prioritized. Voice break was defined as a binary variable (yes/no) according to occurrence of unintentional falsetto notes or voice deepening. Self-reported occurrence was compared to observations during examinations and again the latter was prioritized in case of incongruence.

A wall-mounted stadiometer (Holtain Ltd., Crymych, UK) was used to measure standing height to the nearest 0.1 cm. The boys were weighed on a digital electronic scale (Seca, Hamburg, Germany) to the nearest 0.1 kg while wearing light clothing and no shoes. BMI was calculated as weight divided by height squared (kg/m^2^).

### Reproductive hormone assays

All non-fasting blood samples were drawn between 8:00 AM and 2:00 PM (cross-sectional samples) or 8:00 to 10:00 AM (longitudinal samples) from an antecubital vein, clotted and centrifuged; serum was stored at −20°C until hormone analyses. Serum levels of LH were measured by time-resolved immunofluorometric assays (Delfia; PerkinElmer, Boston, MA, USA) with a detection limit of 0.05 IU/L. Intra- and interassay coefficients of variation (CVs) were <5%. Serum total testosterone was measured by radioimmunoassay using a Coat-A-Count RIA kit (Siemens, Los Angeles, CA, USA) with a detection limit of 0.23 nmol/L. The assay was validated against liquid chromatography coupled with tandem mass spectrometry in *n* = 20 participants (Mouritsen *et al*., 2014). Intra- and interassay CVs were 17% and 12.8%, respectively. LH and total testosterone levels below the detection limit were assigned a value of 0.025 IU/L and 0.115 nmol/L, respectively (0.5 times the detection limit). BMI data of Cohort A (cross-sectional) have previously been reported (Sørensen *et al*., 2010).

### Statistical analyses

We used two approaches to assess the ‘age at a puberty event’. First, in the longitudinal study (Cohort B) we calculated an individual’s age at any puberty event as the mean age between pre- and post-event visits. The same method was used to calculate other values, i.e. reproductive hormones or testicular size, at any puberty event. While examinations were performed around every 6 months, boys were free not to participate in all parts of the examination. In this case individual events were excluded from analysis if the interval between pre- and post-event visits was >2 years or interval censored data were not available. Second, in the larger mixed cross-sectional and longitudinal sample (Cohort A+B), we performed analyses (Aksglaede *et al*., 2008) (SAS: proc lifereg, SAS Institute Inc., Cary, NC, USA) to estimate the population mean age (95% CI) at male pubertal event, bi-testicular volume at event and/or total testosterone above limit of detection (LOD) at event. Proc lifereg allows integrating left-, right- and interval-censored observations and ‘event occurred’ yes/no as the binary response variable. Longitudinal data of children experiencing an event during follow-up were included as interval censored data; as right censored data if a child had not experienced the event at his last examination; as left censored data if a child had experienced the event at his first examination. We calculated sex- and age-specific BMI scores (BMI z-scores, zBMI) by comparison to the World Health Organization (WHO) 2007 reference (de Onis *et al*., 2007). For individuals with longitudinal data available, their mean BMI z-score across all visits was calculated. Data from *n* = 17 visits were excluded from lifereg analysis of zBMI effect due to the missing definition of z-scores in the WHO reference below 5 years and above 19 years of age. To evaluate the effect of zBMI on timing of events, zBMI was introduced into the model as a continuous variable. Individual BMI z-scores in the COPENHAGEN Puberty Study were found to be largely stable throughout puberty (Busch *et al*., 2017). For the association with prepubertal zBMI (subset of samples), zBMI values (baseline zBMI in longitudinal samples) prior to the testicular enlargement were used. Obesity was defined as zBMI >95th percentile and overweight as zBMI >85th and ≤95th percentile. A *P*-value of <0.05 was considered statistically significant. Other aspects of LH and total testosterone levels have previously been published (Sørensen *et al*., 2012).

Individual growth curves were analysed using SuperImposition by Translation and Rotation (SITAR) growth curve analysis (Cole *et al*., 2010) (‘sitar’ package in *R*) using height and logarithmically transformed age to calculate peak height velocity (PHV). Given the limited number of longitudinal samples (*n* = 93), we did not assess the association of zBMI with PHV.

### MR analyses

Two-sample MR analysis was performed to determine the likely causal effect of (genetically determined) BMI (exposure) on (genetically determined) timing of puberty (outcome). For BMI, we used publicly available GWAS summary statistics on 97 variants from the GIANT consortium; in those studies, adult BMI was inverse normally transformed and therefore effect estimates for GWAS variants are quantified as approximate SD (Locke *et al*., 2015). Genetic variants associated with adult BMI were used as exposure due to the strength of the instrumental variable compared to genetic variants associated with childhood BMI. Nevertheless, recent data highlight a strong genetic correlation (*r_g_* = 0.73) between the two traits (Felix *et al*., 2016). GWAS summary statistics for age at voice breaking were from 55 871 male research participants in the 23andMe study. As previously reported in detail (Day *et al*., 2015a), recalled age at voice breaking was based on an online questionnaire using predefined 2-year bins. Inverse-variance weighted models were performed, with additional weighted median and MR-Egger tests (Bowden *et al*., 2015) to exclude violations of the test assumptions, i.e. non-directional and directional pleiotropy.

### Ethical considerations

The COPENHAGEN Puberty Study (ClinicalTrials.gov ID: NCT01411527) was approved by the ethical committee of The Capital Region of Denmark (No. KF01282214) as well as the Danish Data Protection Agency (No. 2015-41-4494). 23andMe participants provided informed consent to take part in this research under a protocol approved by Ethical & Independent Review Services, an institutional review board accredited by the Association for the Accreditation of Human Research Protection Programs.

**Table I:** Timings of puberty events and their associations with zBMI in boys.

Pubertal events	Timing of events				Association with zBMI^e^	
	Longitudinal only *N*	Age at event, yrs (range or SD)^a^	Cross-sectional & longitudinal *N*	Age at event, yrs (95% CI)^b^	Regression coefficient, yrs per zBMI (95% CI)	*P*-value
**Clinical signs**						
Gonadarche	62	11.5 (9.9–13.8)	713	11.6 (11.5–11.7)	−0.32 (−0.44 to −0.20)	<0.001
Testicular enlargement^c^	62	11.6 (9.9–13.8)	714	11.6 (11.5–11.8)	−0.36 (−0.48 to −0.25)	<0.001
Sweat odor	73	11.8 (8.5–14.3)	726	12.4 (12.1–12.6)	−0.47 (−0.65 to −0.29)	<0.001
Pubarche	63	12.0 (9.6–13.8)	713	12.2 (12.1–12.4)	−0.45 (−0.61 to −0.30)	<0.001
Axillary hair growth	57	13.1 (11.5–15.4)	727	13.6 (13.5–13.8)	−0.36 (−0.51 to −0.20)	<0.001
Voice break	55	13.5 (11.9–15.5)	648	13.6 (13.5–13.8)	−0.33 (−0.47 to −0.18)	<0.001
Peak height velocity	93	13.7 (0.94)	-	-	-	-
**Hormones**						
Total testosterone above LOD^d^	63	11.5 (10.0–13.8)	662	11.7 (11.6–11.9)	−0.30 (−0.43 to −0.18)	<0.001

a ^a^Median (range) or mean (SD) for ‘peak height velocity’.

b ^b^Mean/intercept (95% CI) using SAS proc lifereg.

c ^c^Testicular volume ≥4 mL (at least one testis).

d ^d^Limit of detection (LOD) total testosterone: 0.23 nmol/L.

e ^e^Sex- and age-specific BMI scores (BMI z-scores: zBMI) in cross-sectional samples; mean zBMI across all timepoints in longitudinal samples.

**Table II:** Focus on voice break—cross-sectional and longitudinal part.

Pubertal events	Longitudinal only *N*	Median (10th–90th pct.)^a^	Cross-sectional & longitudinal *N*	Value (95% CI)
**Values at voice break**				
Bi-testicular volume	47	24 (12.6–35.6) mL	612	24 (23–25) mL
Serum total testosterone	40	12.0 (3.7–20.1) nmol/L	561	10.9 (10.0–11.7) nmol/L
Serum LH	41	2.7 (1.4–4.1) IU/L	555	2.4 (2.2–2.5) IU/L
**Intervals**				
Testicular enlargement^b^ to voice break	46	2.0 (1.0–3.0) yrs	-	-
Testosterone over LOD^c^ to voice break	48	1.9 (1.0–2.7) yrs	-	-

a ^a^Median (10th–90th percentile) in yrs/volume/concentration.

b ^b^Testicular volume ≥4 mL (at least one testis).

c ^c^LOD: total testosterone: 0.23 nmol/L.

## Results

### Temporal analysis of pubertal milestones including voice break

**Figure 1:**
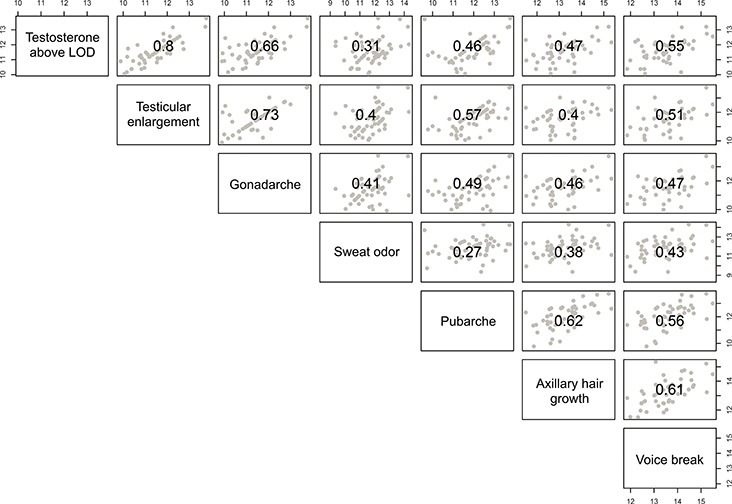
**Scatterplot matrix and correlations of timing of pubertal events in boys (longitudinal samples, *n* = 93).** Pearson correlation coefficients between timing of pubertal events (in years) are indicated in the center of each box while dots represent individual events.

Voice break occurred at mean age of 13.6 (95% CI: 13.5–13.8) years in the combined cross-sectional and longitudinal sample. Voice break and PHV were late events of puberty in boys compared to testicular enlargement, gonadarche, sweat odor, pubarche and axillary hair growth (Table I). Timing of pubertal events was comparable when assessed by compiled data (mixed cross-sectional and longitudinal samples) and longitudinal samples exclusively (Table I). Testicular enlargement ≥4 mL (at least one testis) occurred at 11.6 (95% CI: 11.5–11.8) years and coincided with detectable testosterone in serum (total serum testosterone above LOD) occurring at 11.7 (11.6–11.9) years. Testicular enlargement ≥4 mL and total serum testosterone above LOD occurred at a median of 2.0 (10th–90th percentile: 1.0–3.0) and 1.9 (10th–90th percentile: 1.0–2.7) years prior to voice break, respectively, with consistent results in the mixed cross-sectional and longitudinal as well as longitudinal samples (Tables I and II). Based on individual timing of pubertal milestones among the longitudinal samples, timing of voice break correlated moderately with other milestones; *r^2^* ranging from 0.43 to 0.61 (Fig. 1). The strongest correlation between pubertal milestones was observed between age at testicular enlargement and testosterone above LOD (*r^2^* = 0.80). At voice break, mean (95% CI) circulating testosterone levels, LH levels and bi-testicular volume were 10.9 (10.0–11.7) nmol/L, 2.4 (2.2–2.5) IU/L and 24 (23–25) mL, respectively (mixed cross-sectional and longitudinal samples; Table II). Data in longitudinal samples confirmed these mean values; however, inter-individual variation was large (Table II).

**Figure 2:**
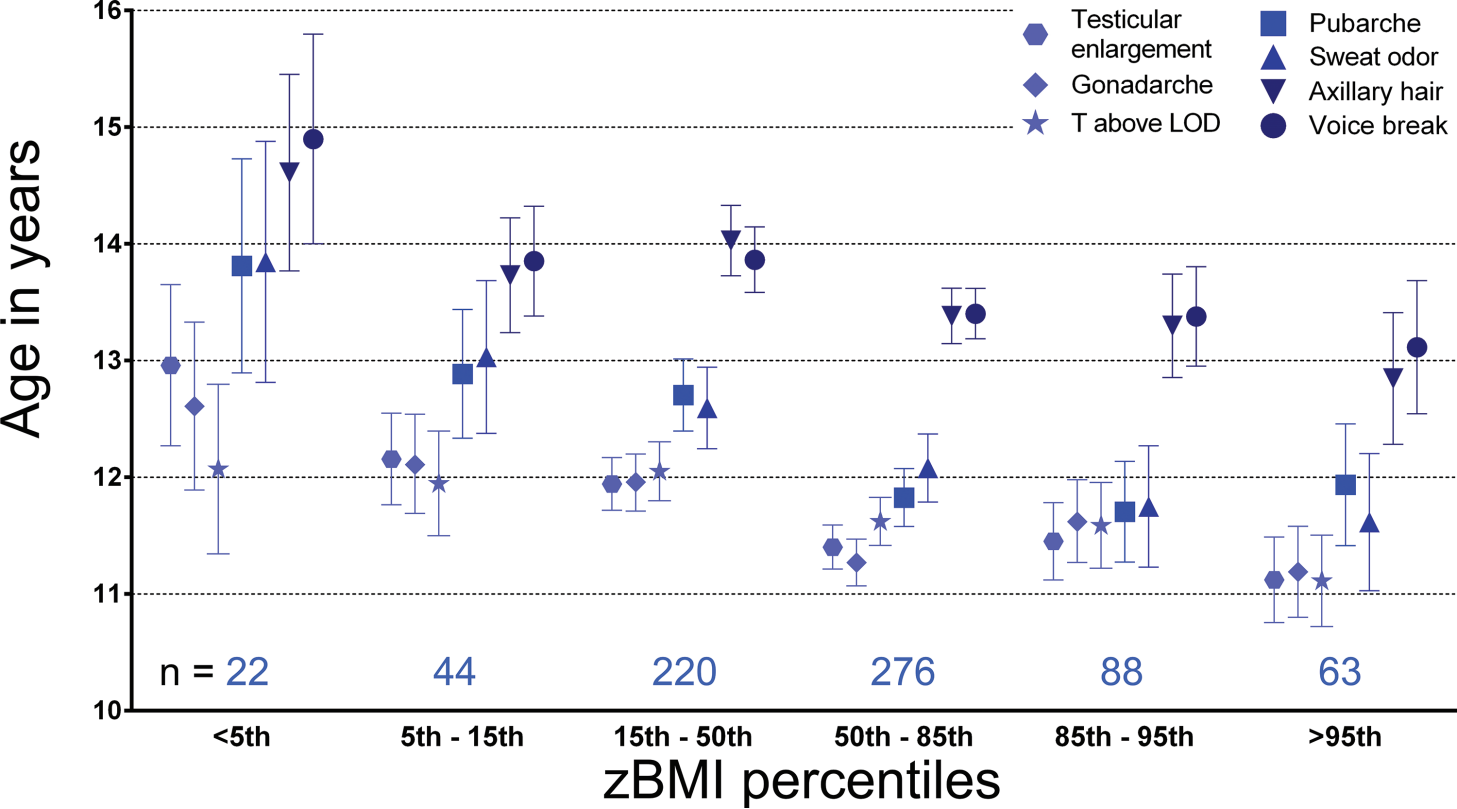
**Timing of pubertal events across the BMI spectrum (percentiles).** Dots/boxes/triangles/stars represent mean age at event-based analysis in both cross-sectional and longitudinal samples. Lines extend to 95% CI. *N* per sex- and age-specific BMI score (BMI z-score: zBMI) group are indicated above the *x*-axis.

### Association of timing of pubertal milestones with BMI

We observed significant negative associations of BMI z-score (zBMI) with timing of male pubertal milestones as well as age at detectable testosterone in serum (all *P* < 0.001 in the combined sample; Table I). We repeated the analysis using prepubertal zBMI (in the subset of boys with prepubertal data) and confirmed significant associations with all milestones (Supplementary Table S1). Effect sizes ranged from −0.33 years (95% CI: −0.47 to −0.18) per zBMI for voice break to −0.47 years (95% CI: −0.65 to −0.29) per zBMI for sweat odor (Table I). All associations with zBMI appeared to be linear with the earliest timing of all pubertal milestones (except for pubarche) seen in obese children (BMI >95th percentile; Fig. 2). Although our approach does not allow for detailed analysis of duration of puberty, it does not appear that the interval from testicular growth to voice break exhibits large differences between zBMI groups (Fig. 2).

### MR analysis: likely causal effect of BMI on puberty timing

**Figure 3:**
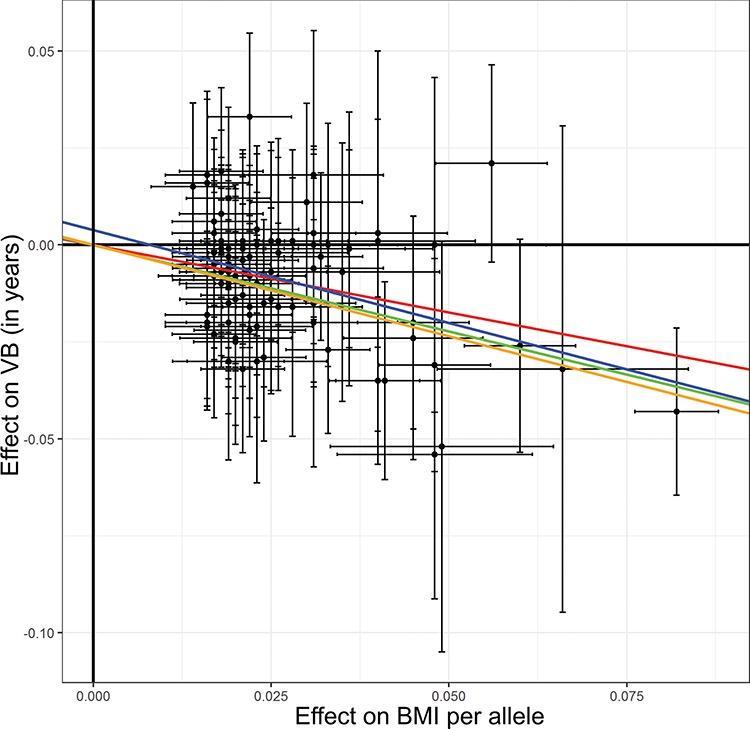
**Dose-response plots for Mendelian randomization analyses of BMI on puberty timing.** Effects of 97 single-nucleotide polymorphisms associated with BMI on age at voice break (VB) in males. Summary statistics were obtained from the 23andMe study (*n* = 55 871 men). Error bars indicate 95% CI for individual single-nucleotide polymorphisms. Slopes indicate Mendelian randomization analyses using inverse variance weighted IVW (red), weighted median (green), EGGER (blue) and penalized weighted median (orange). EGGER: EGGER model. WM: Weighted median model.

Two-sample MR analyses identified likely causal effects of higher BMI on earlier voice break in males (inverse-variance weighted model: −0.35 years per approximate SD, 95% CI: −0.44 to −0.26). The estimate was consistent in sensitivity analyses; i.e. weighted median and Egger’s models (Table III and Fig. 3), suggesting no evidence of horizontal pleiotropy.

**Table III:** Mendelian randomization analysis of the likely causal effects of BMI on voice break.

			Tests of model assumptions				Sensitivity models			
Outcome	Beta_IVW^bc^	95%CI_IVW	Cochrane_Q	Cochrane_P	Intercept_EGGER	95%CI_intercept EGGER	Beta_EGGER	95%CI_EGGER	Beta_WM	95%CI_WM
Age at voice break^a^	−0.348	−0.44 to −0.26	60.3	1	0.004	−0.002 to 0.010	−0.478	−0.73 to −0.23	−0.445	−0.60 to −0.29

a ^a^Data on age at voice break from the 23andMe study (*n* = 55 871 men).

b ^b^Beta: estimated causal effect of BMI (per approximate SD) on puberty timing (years).

c ^c^IVW: Inverse variance weighted model. EGGER: EGGER model. WM: Weighted median model.

## Discussion

In this mixed cross-sectional and longitudinal study of 730 healthy Danish boys, we provide a comprehensive temporal analysis of male pubertal milestones in relation to age of voice break and reproductive hormone dynamics. We confirm voice break as an event in late puberty exhibiting moderately strong correlations with the timing of other milestones. We further report a consistent negative association of age-specific BMI with all male pubertal milestones across the entire zBMI spectrum. Interestingly, using an MR approach based on large-scale genetic data, we report a likely causal effect of BMI on timing of male (voice break) pubertal timing.

In the present study, we found age at voice break, as evaluated by trained physicians, to be moderately strong correlated with other milestones occurring approximately 2 years after clinical onset of puberty (testicular enlargement) and detectable testosterone levels (total testosterone above LOD). We observed a strong correlation of testicular enlargement and rise in testosterone levels highlighting co-occurrence of these events. Voice break occurred at an advanced stage of testicular growth at mean levels of serum total testosterone of around 11 nmol/L. Concerning the link between genital development/testicular enlargement and testosterone levels at voice break, our results are in accordance with previous studies (Lee *et al*., 1974; Pedersen *et al*., 1986; Harries *et al*., 1997). However, results from our longitudinal samples highlight that voice break can occur over a broad range of serum total testosterone levels. Previous studies reported a strong correlation of speaking/singing fundamental frequency with vocal cord length and testicular volume (Harries *et al*., 1998) while correlation with testosterone levels was only moderate (Harries *et al*., 1997). This may be due to diurnal variation in testosterone levels with high levels during the night and low level during daytime, particularly in prepubertal boys (Boyar *et al*., 1974). Thus, current and previous observations point to the importance of testicular growth and testosterone production for voice break, but there appears to be no clear threshold of serum total testosterone for voice break.

While in girls the association between higher BMI and earlier puberty appears to be consistent across studies (Ahmed *et al*., 2009; Aksglaede *et al*., 2009b), the association in boys, particularly in overweight and obese boys, is debated (Ahmed *et al*., 2009; Wagner *et al*., 2012). BMI during childhood and adolescence is strongly correlated with body fat percentage (Wohlfahrt-Veje *et al*., 2014), which is an important central regulator of the pubertal reactivation of the HPG axis (Day *et al*., 2015c). In detailed analysis stratified by zBMI groups, we observed significant associations of higher zBMI with earlier timing of voice break in boys (consistent with previous reports; Juul *et al*., 2007; Ong *et al*., 2012) as well as all other male pubertal milestones. Comparable to our observations, a number of studies report an inverse linear relation between BMI and male pubertal onset across the entire BMI spectrum (Sandhu *et al*., 2006; Juul *et al*., 2007; Aksglaede *et al*., 2009a). However, some studies report later pubertal onset in obese boys compared to overweight boys suggesting a non-linear J-shaped association (Wang, 2002; Lee *et al*., 2010, 2016). In our study, covering the full spectrum of BMI, we observed a linear association of timing of male pubertal milestones with zBMI. In fact, all events (except pubarche) analysed in the mixed cross-sectional and longitudinal part occurred earlier in obese boys compared to overweight boys.

While previous studies, as well as our present study, demonstrate a significant association between puberty timing and BMI, it remains unclear what is cause and what is effect. While the association of prepubertal BMI with pubertal timing is rather consistent with an effect of BMI on puberty timing than vice versa, the pubertal transition is accompanied by alterations in age-specific BMI itself (Ong *et al*., 2012) and reverse causation cannot be excluded using observational data. However, by means of a two-sample MR analysis, we reveal novel and robust evidence for a likely causal effect of higher BMI on earlier voice breaking in males. Notably, the inferred effect of BMI on earlier voice breaking (−0.35 years/approximate SD of BMI) was remarkably similar in magnitude to the direct association in our clinical male cohort. Since the MR approach considered only a linear relationship we could not assess the shape, or possible non-linearity, of the causal association using this approach (e.g. obese versus overweight). Heterogeneity between effects of BMI variants on puberty timing was observed for some variants, exemplified by the missense variant rs2229616 in *MC4R* (coding for Melanocortin 4 receptor) with a large effect on BMI but no significant effect on relative age of voice break. Sensitivity models (i.e. EGGER and Weighted median model) accounting for such heterogeneity produced consistent results.

The primary strength of this study is the assessment of pubertal milestones including voice break by clinical examinations performed by a limited number of trained physicians. Secondly, we reveal cause and effect of BMI and pubertal timing by novel large-scale genetic analyses. We used the advantage of longitudinal samples to calculate individual periods between two pubertal events, as well as data from mixed cross-sectional and longitudinal samples using all censored data. Timing of pubertal milestones in our study is comparable to other contemporary Danish puberty cohorts (Wohlfahrt-Veje *et al*., 2016; Brix *et al*., 2019). While the correlation of voice break and testicular enlargement in our clinical cohort was moderately strong suggesting clinically assessed voice break as a valid proxy for puberty timing, we cannot draw final conclusions on the validity of self-reported voice break used in epidemiological studies. It is noteworthy that the validity of self-reported voice break has never been formally assessed, while self-reported genital development in boys is known to be prone to misclassification, particularly overestimation of the genital development (Rasmussen *et al*., 2015). Further, our study was population-based allowing analysis of associations of timing of pubertal milestones with BMI across the entire BMI spectrum. However, we acknowledge that in our study overweight and obese groups differ from other studies in definition and size. Previous studies used a more severe 97th percentile of BMI for definition of obesity, included larger number of obese participants and observed a different association (Kleber *et al*., 2011). Further, combination of observational data of pubertal milestones in healthy children with large-scale genetic data using MR analyses highlights a likely causality of the observed association between BMI and pubertal onset.

In conclusion, we provide a comprehensive temporal analysis of a range of pubertal events in a large contemporary cohort of healthy boys. We confirm voice break as a late milestone of puberty correlating with timing of other milestones. We further observed a linear and likely causal relationship between higher BMI and earlier pubertal timing in males.

## Acknowledgements

We are grateful for the technical help from the skilled personnel in our lab as well as the participating children and their families in Denmark. We also thank the research participants and employees of 23andMe for making this work possible; members of the 23andMe Research Team: Michelle Agee, Babak Alipanahi, Adam Auton, Robert K. Bell, Katarzyna Bryc, Sarah L. Elson, Pierre Fontanillas, Nicholas A. Furlotte, David A. Hinds, Karen E. Huber, Aaron Kleinman, Nadia K. Litterman, Jennifer C. McCreight, Matthew H. McIntyre, Joanna L. Mountain, Elizabeth S. Noblin, Carrie A.M. Northover, Steven J. Pitts, J. Fah Sathirapongsasuti, Olga V. Sazonova, Janie F. Shelton, Suyash Shringarpure, Chao Tian, Joyce Y. Tung, Vladimir Vacic and Catherine H. Wilson.

## Authors’ roles

A.J., K.S., L.A., K.K.O. and J.R.B.P. designed the study. C.P.H., K.S. and L.A. collected the data. A.S.B., B.H. and F.R.D analysed the data. A.S.B. wrote the paper. All authors participated in critical revision of the manuscript and approved the final manuscript as submitted.

## Funding

This work was supported by Danish Agency for Science, Technology and Innovation (09-067 180); Danish Ministry of the Environment, CeHoS (MST-621-00 065); Capital Region of Denmark (R129-A3966); Ministry of Higher Education and Science (DFF-1331-00 113); Innovation Fund Denmark (InnovationsFonden, 14-2013-4); The International Center for Research and Research Training in Endocrine Disrupting Effects of Male Reproduction and Child Health (EDMaRC). B.H., F.R.D., J.R.B.P. and K.K.O. are supported by the Medical Research Council (MC_UU_12015/2). The 23andMe study is supported by the National Human Genome Research Institute of the National Institutes of Health (R44HG006981).

## Conflict of interest

Members of the 23andMe Research Team are employees of 23andMe, Inc. and hold stock or stock options in 23andMe.

## Supplementary Material

Supp_dez118
