# Supplementary material for: Voice break in boys—temporal relations with other pubertal milestones and likely causal effects of BMI
Source: Hum Reprod. 2019 Jul 26;34(8):1514–22. doi: 10.1093/humrep/dez118 (PMC6688887; doi:10.1093/humrep/dez118)
Supplement: Supp_dez118 [file supp_dez118.pdf]

Human Reproduction, pp. 1–1, 2019
doi:10.1093/humrep/dez118

human reproduction

SUPPLEMENTARY DATA

Supplementary Table S1 **Associations with prepubertal BMI.**

Pubertal events	Cross-sectional & longitudinal N	Association with prepubertal zBMI[c]	
		Regression coefficient, yrs per zBMI (95% CI)	*P*-value
**Clinical signs**			
Gonadarche	394	−0.33 (−0.52 to −0.14)	<0.001
Testicular enlargement[a]	396	−0.39 (−0.56 to −0.22)	<0.001
Sweat odor	393	−0.53 (−0.74 to −0.32)	<0.001
Pubarche	394	−0.52 (−0.73 to −0.31)	<0.001
Axillary hair growth	394	−0.32 (−0.54 to −0.10)	0.004
Voice break	372	−0.26 (−0.49 to −0.03)	0.03
**Hormones**			
Total testosterone above LOD[b]	367	−0.31 (−0.47 to −0.16)	<0.001

[a]Testicular volume ≥4 mL (at least one testis).
[b]Limit of detection (LOD) total testosterone: 0.23 nmol/L.
[c]Sex- and age-specific BMI scores (BMI z-scores: zBMI) in cross-sectional samples; baseline zBMI in longitudinal samples.
